# Design of Fresnel Lens-Type Multi-Trapping Acoustic Tweezers

**DOI:** 10.3390/s16111973

**Published:** 2016-11-23

**Authors:** You-Lin Tu, Shih-Jui Chen, Yean-Ren Hwang

**Affiliations:** Department of Mechanical Engineering, National Central University, Taoyuan 32001, Taiwan; 104323074@cc.ncu.edu.tw (Y.-L.T.); yhwang@cc.ncu.edu.tw (Y.-R.H.)

**Keywords:** acoustic tweezers, Fresnel lens, particle trapping

## Abstract

In this paper, acoustic tweezers which use beam forming performed by a Fresnel zone plate are proposed. The performance has been demonstrated by finite element analysis, including the acoustic intensity, acoustic pressure, acoustic potential energy, gradient force, and particle distribution. The acoustic tweezers use an ultrasound beam produced by a lead zirconate titanate (PZT) transducer operating at 2.4 MHz and 100 V_peak-to-peak_ in a water medium. The design of the Fresnel lens (zone plate) is based on air reflection, acoustic impedance matching, and the Fresnel half-wave band (FHWB) theory. This acoustic Fresnel lens can produce gradient force and acoustic potential wells that allow the capture and manipulation of single particles or clusters of particles. Simulation results strongly indicate a good trapping ability, for particles under 150 µm in diameter, in the minimum energy location. This can be useful for cell or microorganism manipulation.

## 1. Introduction

Contactless micro-particle manipulation in a liquid has drawn considerable interest in many biomedical, biological, and physical applications [1,2,3]. The trapping mechanism employed in contact-less micro-particle manipulation techniques can be hydrodynamic [4], dielectrophoretic [5], optical [6], magnetic, or acoustic [7]. Among these, optical and acoustic techniques are the most common. However, optical tweezers make use of laser light sources which are expensive and may also damage the cells by the generation of heat. Furthermore, the short wavelength of laser light cannot capture large particles, or operate in a medium of high opacity. Acoustic tweezers discriminate between different particles based on their density and compressibility. The long wavelength used by acoustic tweezers can capture a large group of particles at one time. Such a high trapping capacity is essential for certain kinds of practical experiments.

Acoustic tweezers manipulate particles using either a standing wave or a single beam. Early acoustic tweezers utilized two counter-propagating, focused ultrasound beams to produce a standing wave at 3.5 MHz, which could capture a frog’s egg or a latex particle 270 µm in diameter in water [8]. In another application, acoustic tweezers utilized an ultrasound standing wave linear array and a reflector at 2.1 MHz to capture alumina micro-particles with a diameter of 16 µm [9]. In addition, a standing surface acoustic wave (SSAW) focusing technique can be applied to a micro-fluid channel. An interdigital transducer (IDT) deposited on a lead zirconate titanate (PZT) substrate and driven by an AC signal produced a standing wave at the IDT, which allowed the capture of micro-particles at the pressure nodes or antinodes of the standing wave [10]. Standing waves can also be used to levitate particles. An acoustic levitation device, which used a stepping circular vibrating plate and a reflector, could form a standing wave that would levitate a sample and hold it at the pressure node [11]. Ding demonstrated SSAW-based acoustic tweezers that can manipulate particles, cells, or organisms [12].

In contrast, acoustic tweezers based on a single beam use tightly focused transducers to capture micro-particles at the focal point. In an early work, a 30 MHz LiNbO_3_ transducer was used to capture a lipid micro-droplet of a diameter of 126 µm [13]. In another study, acoustic tweezers based on a PZT transducer and an air-reflector Fresnel lens could capture microspheres 70–90 µm in diameter at a frequency of 17.9 MHz [14]. A linear phase array containing 64 elements operated at 26 MHz could capture 45-µm-diameter polystyrene micro-particles [15]. Lam proposed a 200 MHz lensless transducer that can manipulate a single microsphere of 5 or 10 µm in diameter in distilled water [16].

**Figure 1:**
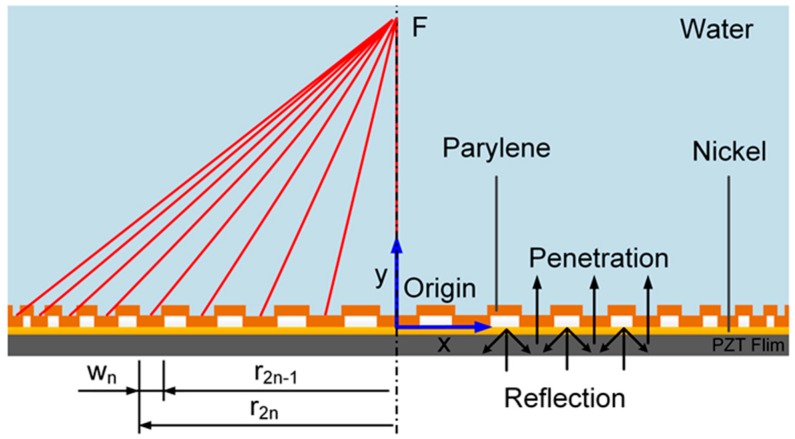
Schematic of the acoustic tweezers with a Fresnel lens. Parylene is deposited on a PZT transducer and patterned according to the FHWB theory. Based on the acoustic impedance theory, the acoustic wave from the PZT transducer transmits through the parylene region to the water and reflects mostly at the air region. The patterned parylene membrane is oriented on the x-axis of the coordinate system.

In this study, acoustic tweezers (Figure 1) built on a PZT transducer with top and bottom electrodes have been proposed. The electrodes were patterned into annular rings to form an acoustic Fresnel lens such that the acoustic waves generated by the annular zones form a beam that has low-energy trapping zones [17]. Some interesting experimental results for particle trapping at the focal plane have been reported in another study by Choe et al. [14]. Our paper aims to simulate the acoustic intensity, acoustic pressure, gradient force, and particle distribution of the acoustic tweezers on the longitudinal plane through the focal point. The finite element method (FEM) was used to conduct numerical simulations of the tweezers’ design which employed acoustic pressure fields produced by air-reflector Fresnel lenses. The simulation results clearly demonstrated that the single beam could effectively capture micro-particles. The simulation results will be beneficial to the future applications and optimization of the acoustic tweezers.

## 2. Theory

In a Fresnel lens design, if the distance from each annular zone to the focal point is a multiple of the wavelength, the acoustic waves will reach the focal point in-phase, or the waves will constructively interfere. This type of acoustic wave source is called the Fresnel half-wave band (FHWB) zone.

The acoustic wavelength in a liquid ($\lambda_{l}$) and the designed focal length (F) determine the diameter of each annular acoustic wave zone. In practice, every annular zone has a radial width (W) and to minimize the destructive wave interference due to the phase variance along the zone width, the radii of the FHWB zone ($r_{k})$ are designed according to [18]

(1) $$r_{k}=\sqrt{2k\lambda_{l}\times \left(F+\frac{k\lambda_{l}}{2}\right)},$$

where *k* = 1,2,3…, n.

According to acoustic impedance theory, an acoustic wave will be reflected largely at the interface of two media if the acoustic impedance of the media differs appreciably. In the simulation, an air-reflector lens was designed by using parylene and air as the media. The acoustic impedance of parylene is similar to that of water and is relatively higher than that of air. Therefore, the acoustic wave produced by the transducer will pass through the parylene layer but reflect at the air region, as shown in Figure 1. The reflectance of the acoustic wave can be calculated by Equation (2), based on which the most suitable design and media for the Fresnel lens can be selected [19].

(2) $$R={\left[\left(Z_{2}/\left(\mathrm{COS}\alpha_{2}\right)-Z_{1}/\left(\mathrm{COS}\alpha_{1}\right)\right)/\left(Z_{2}/\left(\mathrm{COS}\alpha_{2}\right)+Z_{1}/\left(\mathrm{COS}\alpha_{1}\right)\right)\right]}^{2},$$

where $Z_{1}$ and $Z_{2}$ are the acoustic impedances of the two media; $\alpha_{1}$ and $\alpha_{2}$ are the incidence angle and transmission angle.

The acoustic tweezers are immersed in the inviscid working fluid and a sinusoidal signal of angular frequency ω is applied to it. The acoustic beam is time-harmonic and can be represented by the acoustic pressure $P_{in}\left(r\right)e^{-i\omega t}$ and velocity field $V_{in}\left(r\right)e^{-i\omega t}$, both being coordinated at time t and position r. Therefore, the pressure-velocity relation in the first-order approximations can be described in terms of the conservation of the momentum equation as follows [20]:

(3) $$V_{in}=-\frac{i}{\rho_{f}c_{f}k}\nabla P_{in},$$

where $k=\omega /c_{f}$, $\rho_{f}$ is the density, and $c_{f}$ is the acoustic speed in the working fluid. For simplicity, the time-harmonic term $e^{-i\omega t}$ is neglected. Then, the acoustic pressure potential energy can be described as [20]

(4) $$U=V_{0}\left(\frac{{\left|P_{in}\right|}^{2}}{4\rho_{f}{c_{f}}^{2}}f_{1}-\frac{3\rho_{f}{\left|V_{in}\right|}^{2}}{8}f_{2}\right),$$

where $V_{0}$ is the particle volume; $f_{1}$ and $f_{2}$ are the acoustic contrast factors, calculated as

(5) $$f_{1}=1-\frac{\rho c^{2}}{\rho_{f}{c_{f}}^{2}}\text{ and }f_{2}=\frac{2\times \left(\rho -\rho_{f}\right)}{2\rho +\rho_{f}},$$

where ρ and c are the mass density and acoustic speed of the particle, respectively. The gradient force will trap the micro-particle at the minima of the potential energy *U*. This gradient force (F) is calculated by [20]:

(6) F=−∇U.

Therefore, the acoustic trapping force of the gradient force is affected by several parameters such as the acoustic wave frequency, acoustic intensity, particle size, and type of acoustic medium.

## 3. Simulation

The FEM software COMSOL Multiphysics^TM^ was used to simulate the acoustic pressure field. The simulation module included the acoustic pressure, particle tracing for fluid flow, electrostatics, and solid mechanics. The simulation environment was set as an elastohydrodynamic fluid. According to FHWB theory, the dimension of the annular acoustic zone of the air-Fresnel lens should follow Equation (1). 

The piezoelectric transducer element (PZT-5H) was 20 mm in diameter. The host medium was water with the following characteristics: density $\rho_{0}$ = 1000 kg/m^3^, acoustic speed $c_{0}$ = 1481 m/s, kinematic viscosity $\upsilon_{0}$ = 10^−6^ m^2^/s, and acoustic impedance 1.48 MRayls. In the micro-particle distribution simulation, the density of the particle was set to 1000 kg/m^3^ and the diameters used in the simulations were 50, 100, and 150 µm. The material parameters of the air and parylene are listed in Table 1. The voltage and resonance frequency applied to the acoustic tweezers component were 100 V_peak-to-peak_ and 2.4 MHz, respectively. Two-dimensional numerical simulations were performed for the acoustic tweezers. Typically, the Fresnel lens focused on only a single point at a focal length of 10 λ, which was about 6.16 mm along the y-axis.

There are 295,390 nodes used in our simulation. The mesh geometry type used in this paper was free-triangular. For the mesh, the maximum element size was 0.05 mm while the minimum element size was 0.444 µm, which was one-tenth the dimension of the thinnest membrane. The element size range was chosen as a trade-off of the calculation time and the smoothness of the simulation results.

## 4. Results and Discussion

**Figure 2:**
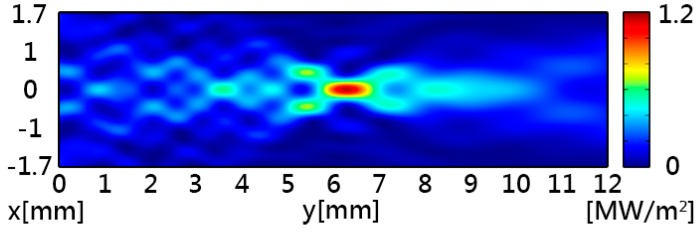
Simulated acoustic intensity of the acoustic tweezers. The highest intensity of 1.15 MW/M^2^ is at about 10 λ focal length in the y-direction.

**Figure 3:**
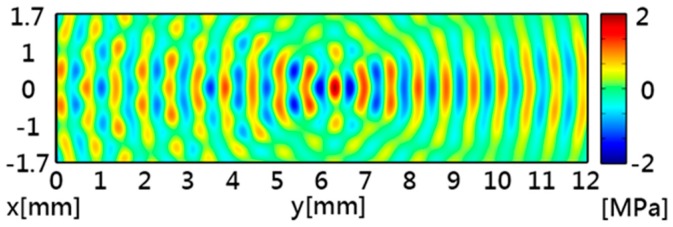
Simulated acoustic pressure of the acoustic tweezers. The maximum acoustic pressure is 1.95 MPa.

**Figure 4:**
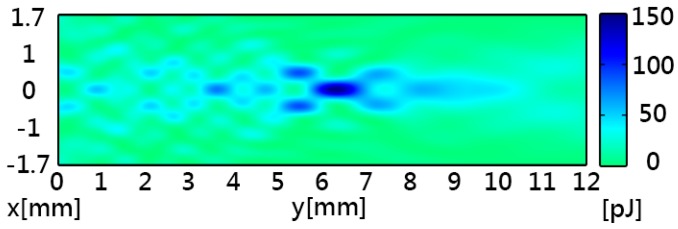
Simulated acoustic potential energy of the acoustic tweezers. The blue regions correspond to high-potential-energy regions, while the green regions correspond to zero-energy regions.

Figure 2 shows the simulated acoustic intensity with a maximum intensity of 1.15 MW/m^2^ at the focal point. Figure 3 shows the simulated acoustic pressure, which had similar distribution patterns to the acoustic intensity. The acoustic potential energy is the crucial characteristic for particle trapping, and it can be calculated by using Equations (5) and (6). In Figure 4, the pattern of potential energy for the acoustic tweezers shows the pattern of the particle distributions. According to the potential well theory, particles will stay in regions of low potential energy which are surrounded by higher-energy regions.

**Figure 5:**
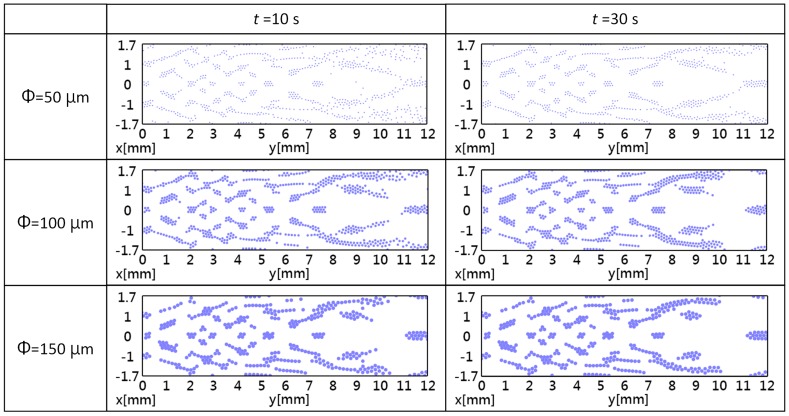
The two-dimensional simulation results of the micro-particle distribution of Fresnel lens acoustic tweezers, where Φ is the particle diameter and *t* is the simulated time.

The two-dimensional simulation results of trapping for the three particle diameters are shown in Figure 5. There was an agreement between the simulated acoustic potential energy and the simulated particle distributions, and the patterns of the retained particles matched the patterns of the low energy regions. The diameter of the particle also influences the particle capture and capture duration. The particle distribution patterns with a small diameter matched the low energy areas better than those with a larger diameter. In addition, it can be observed that the acoustic tweezers have different pattern formation times. In the beginning (*t* = 10 s), only the particles near the Fresnel lens gather in the low-acoustic-potential-energy regions. After 30 s (*t* = 30 s), the particles at any location were able to form a durable pattern. This is caused by energy decay in the water medium: the energy is higher near the PZT transducer. 

**Figure 6:**
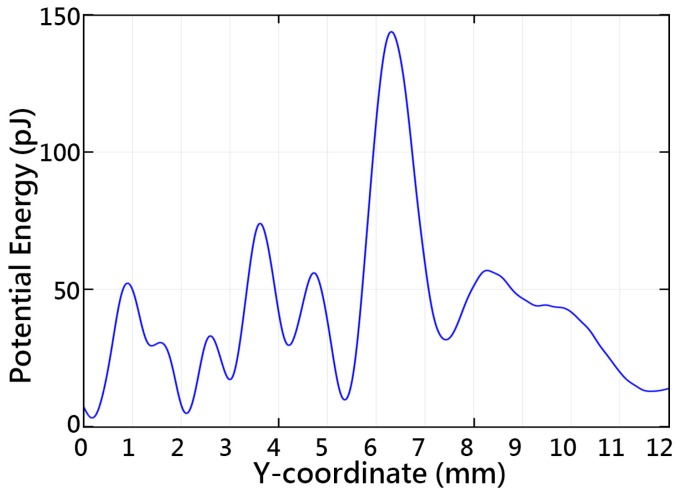
Simulated acoustic potential energy along the central axis of the acoustic tweezers.

**Figure 7:**
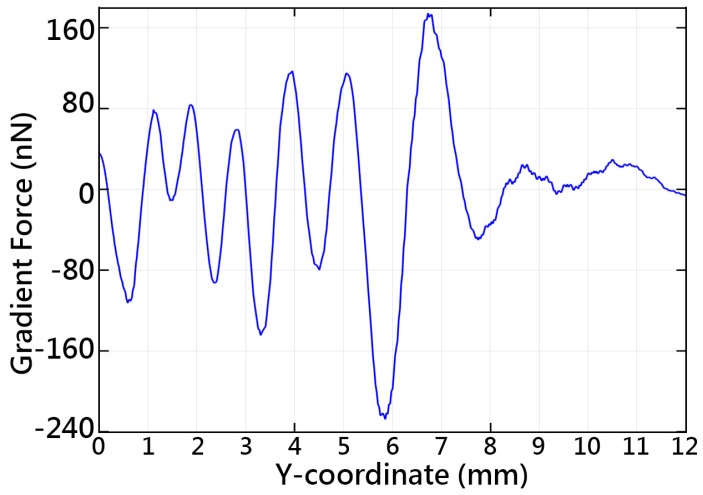
Simulated gradient force along the central axis of the acoustic tweezers.

Along the central axis, the acoustic tweezers gather most of the particles at 4.1–4.35, 5.2–5.6, and 7.3–7.8 mm. However, particles in other locations were gradually pushed to the low-energy areas. There were several low-potential-energy regions that could be used for particle trapping, around 2.1, 3, 4.2, 5.3 and 7.4 mm, as can be seen in Figure 6. There is a similarity between the simulated acoustic potential energy and the particle distribution. Figure 7 shows the simulated acoustic gradient force along the central axis found using Equation (6). The particles are captured in the low-potential-energy regions properly surrounded by high-energy regions. It can be seen that the particles could be retained at locations of zero force which correspond to the extrema of the acoustic forces. The maximum force magnitudes of the tweezers can reach 231 nN.

## 5. Conclusions

Multi-trapping Fresnel lens acoustic tweezers based on PZT transducers are an effective method for simultaneous manipulation of micro-particles. Based on the FEM numerical simulation results, acoustic tweezers operating at a frequency of 2.4 MHz and an input voltage of 100 V_peak-to-peak_ can trap and sustain particles in water. A simulated acoustic field with a maximum intensity of 1.15 MW/m^2^ was observed. The acoustic tweezers have a maximum acoustic pressure magnitude of 1.95 MPa around the focal point. The simulations showed two main regions for particle trapping along the central axis at around 5.3 mm and 7.4 mm. Both regions have wide potential wells compared to others. In some specific low-energy regions, the particles were gradually pushed away to gather in a nearby low-energy region. The trapping force and acoustic potential energy are on the order of nano-newton and pico-joule magnitude. The zero-potential-energy regions correspond to the regions of the extrema of the acoustic gradient forces. This single-beam–based acoustic trapping technique may provide a simple and economical research tool, especially for cell-trapping applications.

## Figures and Tables

**Table 1:** Material parameters.

Parameter	Air	Parylene
Density ($\mathrm{kg}\cdot m^{-3}$)	1.19	1289
Speed of sound ($m\cdot s^{-1}$)	343.2	2142
Acoustic impedance (MRayl)	4.08 × 10^−4^	2.76
